# Prediction of 48-h Intensive Care Unit Mortality in Hospitalized Cancer Patients: Performance of the EPIC Deterioration Index and Sequential Organ Failure Assessment Score

**DOI:** 10.1177/08850666251386399

**Published:** 2025-10-14

**Authors:** Cerena Leung, Lee Cheng, Natalie Walton, Nico Nortje, Kerin Adelson, David Hui

**Affiliations:** 1 Department of Hospital Medicine, 4002 The University of Texas MD Anderson Cancer Center, Houston, TX, USA; 2 Institute of Cancer Care Innovation, 4002 The University of Texas MD Anderson Cancer Center, Houston, TX, USA; 3 Department of Critical Care Medicine, 4002 The University of Texas MD Anderson Cancer Center, Houston, TX, USA; 4 Department of Breast Medical Oncology, 4002 The University of Texas MD Anderson Cancer Center, Houston, TX, USA; 5 Center for Goal Concordant Care Research, 4002 The University of Texas MD Anderson Cancer Center, Houston, TX, USA; 6 Department of Palliative Care, Rehabilitation and Integrative Medicine, 4002 The University of Texas MD Anderson Cancer Center, Houston, TX, USA; 7 Department of General Oncology, 4002 The University of Texas MD Anderson Cancer Center, Houston, TX, USA

**Keywords:** prognostic accuracy, SOFA, EDI, 48-h ICU mortality, medical oncology

## Abstract

**Purpose:**

The EPIC deterioration index (EDI) and the Sequential Organ Failure Assessment (SOFA) scores are commonly used. However, no studies have examined their performance in hospitalized cancer patients just before intensive care unit (ICU) transfer. We examined the prognostic accuracy of the EDI and SOFA for 48-h ICU mortality.

**Methods:**

This study evaluated consecutive hospitalized medical oncology patients transferred to the ICU from January 1, 2021 to December 31, 2023. Univariable and multivariable logistic regression models examined if EDI scores (EDI-now) and SOFA scores immediately prior to ICU transfer were predictive of 48-h ICU mortality. We assessed the performance of EDI-now, EDI at 24 h before ICU transfer (EDI-24 h), the difference between EDI-24 h and EDI-now (EDI-change) and SOFA using the area under the receiver operating characteristic curve (AUROC).

**Results:**

There were 1987 hospitalizations among 1907 unique patients (mean age = 61, male = 58%); 236 (11.9%) died within 48 hours of ICU transfer. In the multivariable analysis, EDI-Now (OR [95% CI]; 3.11 [2.26,4.27], p < 0.0001) and SOFA (OR [95% CI]; 1.78 [1.31,2.43], p = 0.0002) were significantly associated with 48-h ICU mortality. The AUROC for EDI-now, EDI-24 h, EDI-change and SOFA were 0.657, 0.563, 0.635, and 0.621, respectively. The optimal cut-off threshold for EDI-now was 55 with 73.7% sensitivity, 54.4% specificity, 17.9% positive predictive value (PPV) and 91.5% negative predictive value (NPV). The optimal cut-off threshold for SOFA score was 5 with 63.1% sensitivity, 53.6% specificity, 15.5% PPV and 91.5% NPV.

**Conclusion:**

EDI-now ≥ 54.3 and SOFA ≥ 4 were associated with greater 48-h ICU mortality. High NPVs and low PPVs suggest EDI and SOFA scores could potentially be used to identify patients at lower risk of dying within 48 hours but cannot be used to predict higher risk of 48-h mortality prior to ICU transfer. Better prognostic scores are needed for the medical oncology population.

## Introduction

Cancer patients are often hospitalized for complications related to disease progression, treatment related complications or worsening of comorbidities as a result of cancer or its treatment.^1,2^ In addition, cancer patients are at higher risk of becoming critically ill as a consequence of multiorgan failure in the setting of anti-neoplastic treatments and immunosuppression.^3–5^ Prior studies report intensive care unit (ICU) mortality rates ranging between 25–50% in the cancer population with a median ICU length of stay (LOS) of 4 days among medical oncology patients who died in the ICU.^6–9^ ICU mortality within 48 hours may suggest poor quality of end of life care and transferring patients at high risk of 48-h ICU mortality may be costly and ultimately futile.

To identify patients at high mortality risk, EPIC automatically generates the Sequential Organ Failure Assessment (SOFA) score daily in which higher scores have been associated with overall hospital mortality and overall ICU mortality within the medical oncology population.^8,10,11^ More recently, EPIC developed the EPIC deterioration index (EDI) score which is automatically generated through proprietary machine learning. Because of the lack of evidence and established cutoff, there is high level of variability in how clinicians may interpret and apply the EDI in clinical practice.^12–14^ Some clinicians have used the EDI score to identify patients at higher risk of mortality and to initiate goals of care discussions or palliative care referrals. However, few studies have evaluated potential predictive value of the EDI for clinical deterioration such as ICU transfer, mechanical ventilation or death in the medical population, COVID, and trauma patients.^12,13,15^ To our knowledge, there are no validated prognostic scores which predict 48-h ICU mortality, and no studies have examined predictors for 48-h ICU mortality in medical oncology patients. If clinicians are able to accurately identify patients who are at high risk of 48-h ICU mortality and may not benefit from ICU transfer, alternatives to the ICU such as the palliative care unit (PCU) could be considered as life prolonging measures would not be appropriate in this setting. Understanding the prognostic significance of the EDI and SOFA scores can potentially improve resource utilization and promote goal concordant care. In this study, we examine the prognostic accuracy of the EDI and SOFA scores for mortality within 48 hours of ICU transfer among medical oncology patients at a comprehensive cancer center.

## Methods

### Design

This retrospective study examined the prognostic accuracy of the EDI and SOFA scores in predicting ICU mortality within 48 hours of ICU transfer among medical oncology patients admitted to The University of Texas MD Anderson Cancer Center (MDACC) between January 1, 2021, and December 31, 2023. The Institutional Review Board at MDACC approved this study and provided a waiver for informed consent.

### Eligibility Criteria

Consecutive adult patients (aged >18 years old) admitted through the MDACC emergency department to a medical inpatient team in Houston, Texas, United States during the study period were eligible for this study. All patients must have an admission order to the general ward placed at least 24 hours prior to a transfer order to the ICU. If a patient required multiple ICU transfers during one hospital encounter, only the first ICU transfer that occurred after a continuous 24 h LOS on the general ward during the hospital encounter was included for statistical analysis. Patients who were transferred from an outside facility to the general ward at MDACC or directly admitted to the ICU were excluded.

### Data Collection

Data was obtained from the electronic health record (EHR). Patient demographics and clinical outcomes were obtained including patient age, sex, race, cancer site, admission type, time to ICU transfer, overall hospital LOS, overall ICU LOS, and discharge disposition.

The EDI was automatically generated every 15 min in the EHR throughout each hospitalization encounter. The total score ranged from 0 to 100. Clinical variables that were contributing factors as well as their weighted contribution were determined through proprietary machine learning and differed among patients. Variables commonly contributing to the total EDI score included but were not limited to the patient age, systolic blood pressure, respiratory rate, oxygen saturation, heart rate, white blood cell count, platelet count, sodium level, and potassium level.

Because it is unclear what timepoint is the most predictive, we selected 3 different variations of the EDI score for analysis. As one prior study showed that the EDI score's predictive value remained high up to 72 h prior to death within the medical population, this study selected the EDI score generated 24 h prior (EDI-24 h) and the most recent EDI score generated prior to the ICU transfer order (EDI-now) being written to evaluate its prognostic accuracy for 48-h ICU mortality.^12^ The change in value between EDI-24 h and EDI-now (EDI-change) was also evaluated for its ability to predict mortality within 48 h of ICU transfer as the change in EDI score has been shown to be potentially predictive of other adverse outcomes.^15^

The SOFA score was automatically generated in the EHR at midnight every 24 hours throughout each hospital encounter. The total score ranged from 0 to 24. The worst values for each of the 6 clinical variables (respiratory, coagulation, liver, cardiovascular, central nervous system, and renal) that comprise the total SOFA score obtained during the most recent 24 hour period were used to calculate the total SOFA score for each calendar day.^16^ The most recent SOFA score generated prior to the ICU transfer order being placed was used for statistical analysis.

### Statistical Analysis

We used the Chi-square analysis test to evaluate differences in the distribution of categorical variables and the student t-test to compare differences in continuous variables between groups. Continuous variables were expressed as means and standard deviations (SD) or medians. The Kruskal-Wallis test was used to test whether the median is the same between groups. The level of significance used for all tests was a two-sided P-value less than 0.05. The end point measure, death within 48 hours of ICU transfer from the general ward, was evaluated using multivariable logistic regression models to calculate odds ratios (ORs) adjusting for patient's age, sex, race, cancer type, hospital admission type, time to ICU transfer, EDI-now, and SOFA. Because all three variations of the EDI score were strongly correlated with one another, only the EDI-now was included in the multivariate analysis.

The area under the receiver-operating characteristic curve (AUROC) was used to measure the ability of the EDI score and SOFA score to discriminate the risk of mortality between patients who died and survived within 48 hours of ICU transfer. The AUROC was calculated using EDI-now, EDI-24h, EDI-change and the most recent SOFA score generated prior to the ICU transfer order.

Sensitivity, specificity, positive predictive value (PPV) and negative predictive values (NPV) were calculated across a spectrum of SOFA and EDI thresholds. Youden's J statistic was applied to identify a clinically actionable threshold for both SOFA and EDI.

A P-value of 0.05 or lower was considered to be statistically significant. All statistical analysis was performed by using SAS software version 9.4 (SAS Institute, Cary, NC, USA).

## Results

### Patient Characteristics

**Figure 1.**
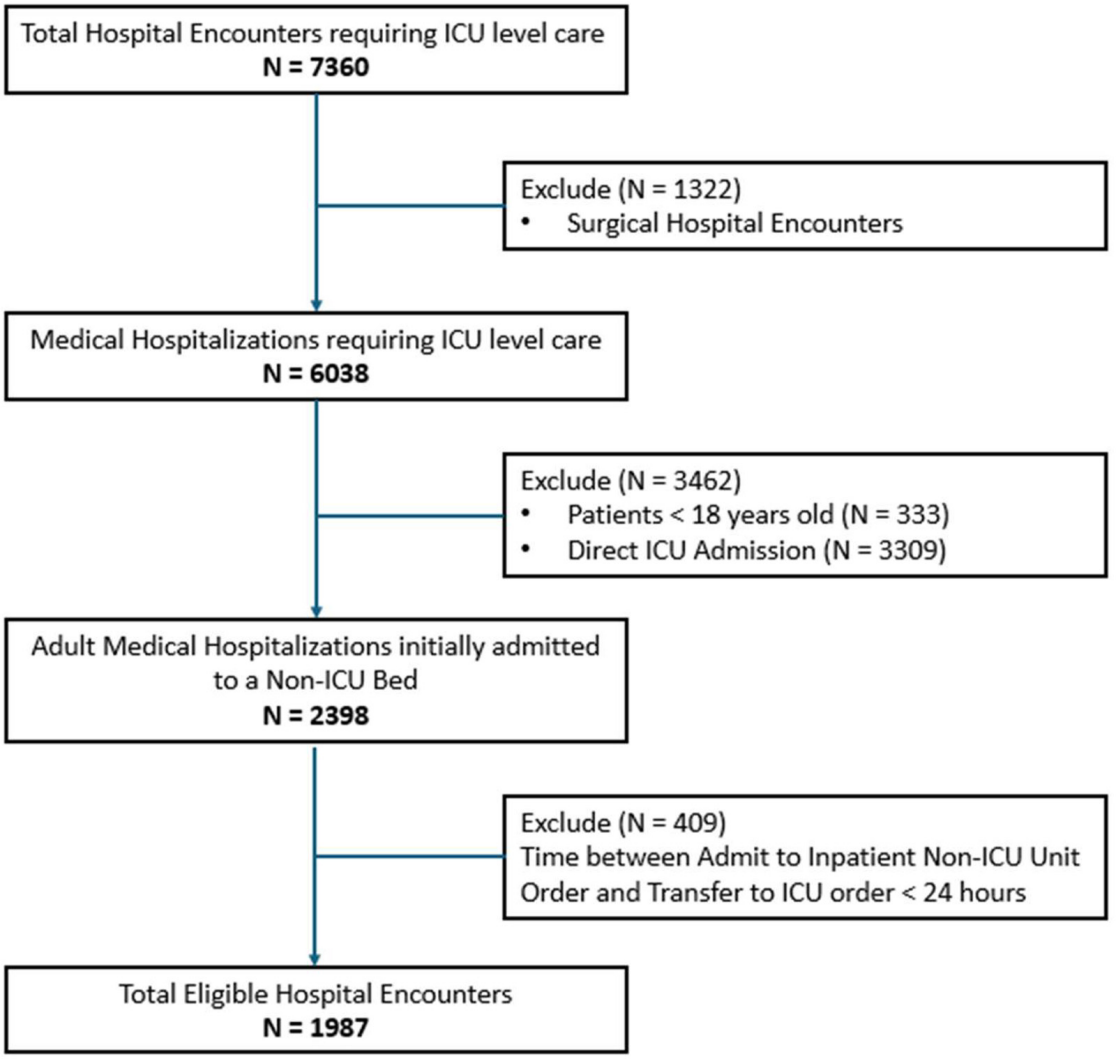
Eligible hospitalization encounters during the study period.

Abbreviations: ICU: Intensive Care Unit.

This study included 1987 hospital encounters among 1907 unique medical oncology patients who were admitted to MD Anderson Cancer Center through the emergency department and required ICU transfer during the hospitalization between January 1, 2021, and December 31, 2023 (Figure 1). The mean age during this study period was 61 years (SD 15) (Table 1). The majority of the patient population was male (58.2%) and white (58.7%), and the three most common primary cancer types were lymphoid, hematopoietic, and myelodysplastic (44.8%); gastrointestinal (11.2%) and thoracic (11.2%) (Table 1). There were 236 (11.9%) hospital encounters resulting in death within 48 hours of ICU transfer (Table 2).

**Table 1.** Patient Characteristics.

Patient Characteristics	Unique Patients N = 1907 (%)
Age, mean (SD)	60.96 (15.2)
Sex	
Male	1109 (58.2)
Female	798 (41.9)
Race	
White or Caucasian	1119 (58.7)
Black or African American	286 (15.0)
Hispanic/Latino	163 (8.6)
Asian	109 (5.7)
Other/Unknown	230 (12.1)
Cancer Site	
Bone, Cartilage, Mesothelial, Skin, and Soft Tissue	90 (4.7)
Breast	67 (3.5)
Gastrointestinal	213 (11.2)
Genitourinary organs	123 (6.5)
Oropharynx	64 (3.4)
Lymphoid, hematopoietic, and Myelodysplastic Syndromes	855 (44.8)
Thoracic organs	214 (11.2)
Endocrine glands	22 (1.2)
Other	259 (13.6)

**Table 2.** Clinical Characteristics by 48-h ICU Survival.

Clinical Characteristics	ICU Transfers		p-value
	48-h ICU Survivor N = 1751 (%)	48-h ICU Non-Survivor N = 236 (%)	
Age, mean (SD)	60.9 (15.3)	60.3 (15.0)	.558
Sex			.068
Male	1011 (57.7)	151 (64.0)	
Female	740 (42.3)	85 (36.0)	
Race			.695
White or Caucasian	1025 (58.5)	139 (58.9)	
Black or African American	269 (15.4)	30 (12.7)	
Hispanic/Latino	147 (8.4)	25 (10.6)	
Asian	103 (5.9)	13 (5.5)	
Other/Unknown	207 (11.8)	29 (12.3)	
Cancer Site			.040
Bone, Cartilage, Mesothelial, Skin, and Soft Tissue	79 (4.5)	14 (5.9)	
Breast	60 (3.4)	8 (3.4)	
Gastrointestinal	174 (9.9)	41 (17.4)	
Genitourinary	114 (6.5)	16 (6.8)	
Oropharynx	60 (3.4)	5 (2.1)	
Lymphoid, hematopoietic, Myelodysplastic Syndromes	809 (46.2)	93 (39.4)	
Thoracic	195 (11.1)	27 (11.4)	
Endocrine	18 (1.0)	4 (1.7)	
Other	242 (13.8)	28 (11.9)	
Index Admission Type			.040
Elective	389 (22.2)	41 (17.4)	
Emergency	901 (51.4)	142 (60.2)	
Urgent	461 (26.3)	53 (22.5)	
Time to ICU Transfer (days), Median (IQR)	6.9 (3.3-13.6)	8.4 (3.9-15.9)	0.024
ICU LOS (Days), Median (IQR)	4.2 (2.2-9.5)	0.8 (0.4-1.37)	<.0001
Overall Hospital LOS (days), Median (IQR)	21.2 (23.9-35.3)	9.4(4.9-17.1)	<.0001
Discharge Disposition			<.0001
Home	520 (29.7)	0 (0.0)	
Home with Home-Health or Physical Therapy	172 (9.8)	0 (0.0)	
Hospice	173 (9.9)	0 (0.0)	
Outside Institution	69 (3.9)	0 (0.0)	
Left Against Medical Advice	6 (0.3)	0 (0.0)	
Rehab Facility, SNF, LTAC	94 (5.4)	0 (0.0)	
In-Hospital Death	717 (41.0)	236 (100.0)	
EDI Score prior to ICU Transfer, Mean ± SD			
EDI-Now	53.49 ± 17.29	62.63 ± 15.10	<.0001
EDI-24h	40.88 ± 13.58	43.39 ± 12.89	.008
EDI-change	12.60 ± 14.78	19.25 ± 14.68	<.0001
SOFA Score, Mean ± SD	4.46 ± 2.76	5.69 ± 2.79	<.0001

Abbreviations: EDI: Epic Deterioration Index; ICU: Intensive Care Unit; LOS: Length of Stay; LTAC: Long Term Acute Care; SNF: Skilled Nursing Facility; SOFA: Sequential Organ Failure Assessment

### EDI Score

The mean EDI-now was 53.5 ± 17.3 versus 62.6 ± 15.1, p < 0.001, and the mean EDI-24 h was 40.88 ± 13.58 versus 43.39 ± 12.9, p = .008, in the 48-h survivor and non-survivor groups, respectively (Table 2). The non-survivors had a significantly larger EDI-change (p < 0.0001) (Table 2). In the multivariate analysis, higher EDI-now remained independently significantly associated with 48-h ICU death (OR [95% CI] 3.11 [2.26, 4.327]) (Table 3).

**Table 3.** Predictors of 48-h ICU Mortality.

	48-h ICU mortality		
Variables	Odds Ratio	95% CI	P-Value
Age			
<50	Reference	Reference	Reference
50–59	1.40	0.90–2.17	0.127
60–69	0.72	0.47–1.09	0.124
70–79	0.79	0.51–1.23	0.304
≥ 80	1.00	0.52–1.89	0.999
Sex			
Female	0.69	0.50–0.96	0.069
Race			
White	Reference	Reference	Reference
Hispanic	1.11	0.68–1.80	0.410
Black	0.72	0.46–1.12	0.149
Asian	0.76	0.41–1.43	0.666
Other	0.97	0.62–1.53	0.924
Admission Type			
Elective	Reference	Reference	Reference
Urgent	0.93	0.58–1.50	0.794
Emergency	1.33	0.88–2.00	0.170
Cancer Site			
Lymphoid, hematopoietic, Myelodysplastic Syndromes	Reference	Reference	Reference
Bone, Cartilage, Mesothelial, Skin, and Soft Tissue	2.06	1.07–3.95	0.030
Breast	1.82	0.78–4.24	0.164
Gastrointestinal	2.53	1.62–3.95	<.0001
Genitourinary	1.47	0.81–2.67	0.198
Oropharynx	0.95	0.35–2.52	0.919
Thoracic	1.40	0.85–2.31	0.179
Endocrine	2.23	0.70–7.09	0.173
Other	1.04	0.66–1.66	0.843
Time to ICU Transfer (days)			
≥ 7.03 (Median)	1.22	0.90–1.65	0.188
EDI-now			
≥ 54.3 (Mean)	3.11	2.26–4.27	<.0001
SOFA			
≥ 4 (Mean)	1.78	1.31–2.43	.0002

Abbreviations: EDI, EPIC Deterioration Index; ICU, Intensive Care Unit; SOFA, Sequential Organ Failure Assessment

**Figure 2.**
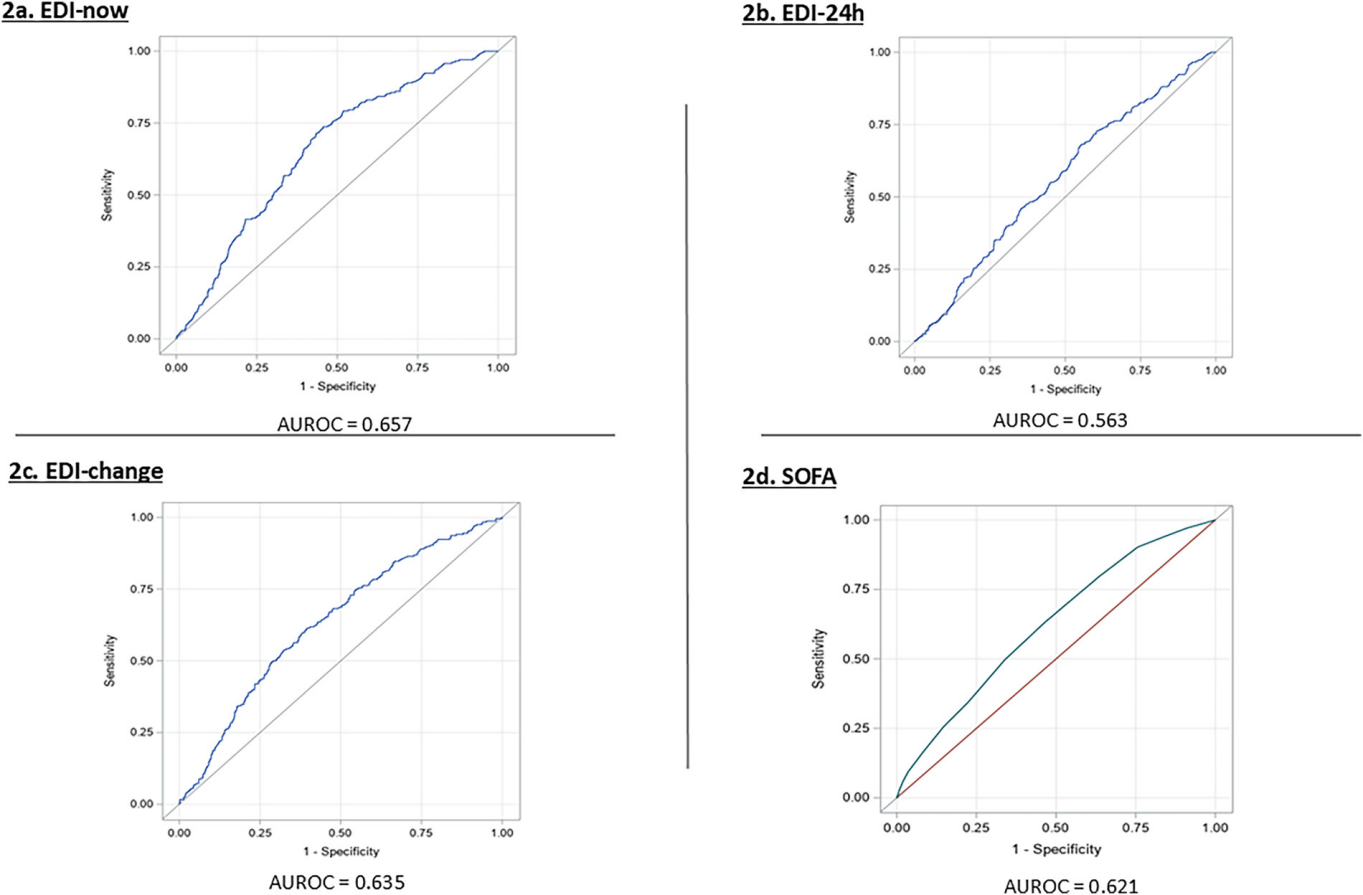
Predictive accuracy of the EDI and SOFA scores for 48-h ICU mortality.

Abbreviations: EDI: Epic Deterioration Index; ICU: Intensive Care Unit; SOFA: Sequential Organ Failure Assessment.

The AUROC of EDI-now and EDI-24h were 0.657 and 0.563, respectively, for 48-h ICU mortality (Figure 2a and b). The AUROC of the EDI-change was 0.635 for 48-h ICU mortality (Figure 2c).

The optimal cut-off threshold identified for EDI-now was 55 with 73.7% sensitivity, 54.4% specificity, 17.9% PPV and 91.5% NPV (Table 4).

**Table 4.** Performance of EDI-now and SOFA with Different Cutoff Points.

EDI-Now	Sensitivity	Specificity	PPV	NPV	Overall accuracy	Youden's J Statistic
>50	0.81	0.44	0.16	0.94	0.49	0.25
55	0.74	0.54	0.18	0.94	0.57	0.28
>60	0.58	0.64	0.18	0.92	0.64	0.22
>70	0.36	0.81	0.20	0.90	0.76	0.17
>80	0.12	0.92	0.17	0.89	0.83	0.04

SOFA	Sensitivity	Specificity	PPV	NPV	Overall accuracy	Youden's J Statistic
>4	0.80	0.36	0.14	0.93	0.42	0.16
5	0.63	0.54	0.15	0.92	0.55	0.17
>6	0.50	0.66	0.17	0.91	0.64	0.16
>8	0.25	0.85	0.19	0.89	0.78	0.11
>10	0.09	0.96	0.26	0.89	0.86	0.06

Abbreviations: EDI: Epic Deterioration Index; NPV: Negative Predictive Value; PPV: Positive Predictive Value; SOFA: Sequential Organ Failure Assessment.

### SOFA Score

The mean SOFA score was 4.46 ± 2.76 and 5.69 ± 2.79, (p < 0.0001) in the 48-h survivor and non-survivor group, respectively (Table 2). In the multivariate analysis, higher SOFA scores generated just prior to ICU transfer remained independently significantly associated with 48-h ICU death (OR [95% CI] 1.78 [1.31, 2.43]) (Table 3).

The AUROC of the most recent SOFA score generated prior to ICU transfer was 0.621 for 48-h ICU mortality (Figure 2d). The optimal cut-off threshold identified for the most recent SOFA score calculated prior to ICU transfer was 5 with 63.1% sensitivity, 53.6% specificity, 15.5% PPV and 91.5% NPV (Table 4).

## Discussion

This study found that a substantial minority (11.9%) of hospital encounters requiring ICU transfer died within 48 h, highlighting the need to identify such patients who may be less likely to benefit from intensive care. We found that higher EDI and SOFA scores were associated with greater 48-h ICU mortality in both the univariable and multivariable analyses; however, their performances were suboptimal with relatively low AUC, low sensitivity, and low positive predictive value, suggesting that it may be difficult to apply these scores in clinical practice to identify at risk individuals. Further research is needed to develop and validate prognostic models to support real-time clinical decision making.

To our knowledge, no prior studies have examined EDI and SOFA scores immediately prior to ICU transfer to identify patients who may not benefit from ICU admission. Existing literature has mostly evaluated prediction models for overall ICU mortality rates based on variables after ICU admission, and few have focused on the medical oncology population. In these studies, 72 hours has been recognized as early ICU death as trends in organ failure during the first 72 hours of an ICU admission have been shown to be predictive of overall death.^17–20^ One prior study reported a 72-h ICU mortality rate of 17.2% in general medicine patients with septic shock and identified age, malignancy, diabetes mellitus, lack of pathogenic identification and higher SOFA scores upon ICU admission as independent predictors of early ICU mortality.^17^ More recently, another study reported a 48-h ICU mortality rate of 5.42% in critically ill patients with sepsis.^21^ Our study found a higher 48-h ICU mortality rate than Driessen et al, though this is not surprising as active malignancy was found to be significantly associated with 48-h ICU mortality.^21^ As such, the ability to predict early ICU mortality prior to ICU transfer, especially in the medical oncology population, would be beneficial as this can prompt earlier goals of care discussions by providers and allow patients the opportunity to participate in these discussions, to ensure goal concordant care is provided and to potentially avoid futile extraordinary life-prolonging care at the end of life.

This study examined the predictive accuracy of 3 different versions of the EDI score for 48-h ICU mortality in the setting of limited available literature. The EDI-now, EDI-24 h, and EDI-change all performed similarly to the SOFA score in predicting 48-h ICU mortality. However, the EDI-now performed slightly better. Though widely available for use, there have only been 6 studies evaluating the prognostic accuracy of the EDI score within the general medical population, COVID patients and trauma patients.^12,13,16,22–24^ These prior studies do report acceptable prognostic accuracy of the EDI score in predicting overall in-hospital death and escalation of care including ICU transfer, cardiac arrest, mechanical ventilation with lead times spanning from 3 hours to 72 hours prior to the primary outcome.^12,13,15,22–24^ Our analysis demonstrated low sensitivity and low positive predictive values indicating that a majority of patients did not die within 48 hours of ICU transfer and that higher EDI scores would not be able to accurately identify the majority of patients at risk of early ICU death. However, high NPVs suggest that clinicians could potentially use low EDI scores to identify patients at lower risk of dying within 48 hours and perhaps more appropriate for ICU transfer. This study highlights that although the EDI score is widely available, clinical utility in predicting 48-h ICU mortality and to ultimately guide decisions regarding ICU transfer remains unclear as this score was not specifically designed for cancer patients or early ICU mortality. Patient and clinical characteristics specific to cancer patients may be unaccounted for in the EDI score, and further study is required to evaluate its utility in other populations and circumstances though this may be difficult given the proprietary machine learning aspect of the EDI score. Other prognostic models may need to be developed and perhaps incorporate physical signs previously shown to occur in advanced cancer patients during the last 3 days of life, including respiration with mandibular movement, peripheral cyanosis, or death rattle.^25^

The SOFA score was initially developed to define the severity of multiorgan failure in critically ill patients and has since become a widely used validated prognostic model to predict multiple outcomes including overall mortality in critically ill patients upon admission to the ICU.^16^ The use of the SOFA score to assess overall ICU mortality has been validated in adult populations in medical-surgical intensive care units with specific conditions such as sepsis, post cardiac arrest, COVID-19 pneumonia, as well as surgical and trauma patients amongst others.^16,26–28^ Modified versions have been created to better prognosticate for various patient populations with specific health conditions, including hematologic malignancies (SOFA-HM).^29^ Similarly, Nates et al ***,*** found that the modified SOFA (mSOFA) was equally predictive of mortality in critically ill cancer patients.^30^ Though our study found that SOFA scores were statistically significantly higher in the 48-h non-survivor group, further analysis demonstrated low sensitivity and low PPVs indicating that high SOFA scores would not be able to accurately identify the majority of medical oncology patients at risk of early ICU death. Specifically, a majority of patients with higher SOFA scores did not die within 48 h of ICU transfer. Similar to the EDI score, high NPVs may suggest that clinicians could potentially use low SOFA scores to identify patients at lower risk of 48-h ICU mortality and therefore more appropriate for ICU transfer. Our study highlights that clinical utility of the SOFA score in predicting early ICU mortality in the medical oncology population prior to ICU transfer is unclear. There may be unique clinical factors specific to the medical oncology population that are unaccounted for in the SOFA score. Further investigation is needed to evaluate the SOFA score's utility in other populations and outcomes.

This study has several limitations. Data was obtained from a single tertiary cancer care center and may not be generalized in other settings. Future studies should consider involving multiple sites to enhance external validity. Due to the retrospective nature of this study, the performance of the EDI and SOFA scores were only evaluated in patients who were transferred to the ICU during their hospitalization. Further studies are needed to further evaluate the prognostic accuracy of the EDI and SOFA scores for 48-h ICU mortality specifically within the surgical oncology, solid tumor and hematologic malignancy population. Though the EDI scores were automatically generated within the electronic health record throughout each hospital encounter, the scores were calculated via proprietary machine learning such that the full list of variables, the parameters of the variables, and the contribution of each of the variables within the prediction model and prior internal validation are not available for review. Additional studies can consider evaluating the predictive value of each component of the SOFA for 48-h ICU mortality. This study did not include prognostic variables such as vital signs, dyspnea or delirium in the multivariable analysis. Reasons for ICU transfer or specific organ failure present on ICU transfer were not included in the analysis, but examination of their predictive value of 48-h ICU mortality can be considered in future studies. Because this study only included patients who were transferred to the ICU, there may be a certain extent of selection bias as patients who were recognized to be actively dying or otherwise deemed inappropriate for ICU transfer were excluded.

## Conclusion

In summary, our study highlights the need to examine the utility of predictive models in the medical oncology population prior to ICU level care. Wide availability of the EDI and SOFA scores may tempt reliance among providers. While these scores may have some prognostic utility, the low AUROC and PPV limit their utility to identify medical oncology patients who may not benefit from ICU transfer. Further study is needed to identify variables specific to the medical oncology population and to create a prognostic model with acceptable predictive value for 48-h ICU mortality in cancer patients.

## References

[1] Manzano JG Luo R Elting LS George M Suarez-Almazor ME . Patterns and predictors of unplanned hospitalization in a population-based cohort of elderly patients with GI cancer. J Clin Oncol. 2014;32(31):3527-3533. 25287830 10.1200/JCO.2014.55.3131 PMC4209103

[2] Hassett MJ Rao SR Brozovic S , et al. Chemotherapy-related hospitalization among community cancer center patients. Oncologist. 2011;16(3):378-387. 21349949 10.1634/theoncologist.2010-0354 PMC3228109

[3] Seo SK Liu C Dadwal SS . Infectious disease complications in patients with cancer. Crit Care Clin. 2021;37(1):69-84. 33190776 10.1016/j.ccc.2020.09.001 PMC8294629

[4] Bos MM Verburg IW Dumaij I , et al. Intensive care admission of cancer patients: A comparative analysis. Cancer Med. 2015;4(7):966-976. 25891471 10.1002/cam4.430 PMC4529335

[5] Martos-Benítez FD Soler-Morejón CD KX L-P , et al. Critically ill patients with cancer: A clinical perspective. World J Clin Oncol. 2020;11(10):809-835. 33200075 10.5306/wjco.v11.i10.809 PMC7643188

[6] Al-Zubaidi N Shehada E Alshabani K ZazaDitYafawi J Kingah P Soubani AO . Predictors of outcome in patients with hematologic malignancies admitted to the intensive care unit. Hematol Oncol Stem Cell Ther. 2018;11(4):206-218. 29684341 10.1016/j.hemonc.2018.03.003

[7] Wei M Huang M Duan Y , et al. Prognostic and risk factor analysis of cancer patients after unplanned ICU admission: A real-world multicenter study. Sci Rep. 2023;13(1):22340. 38102299 10.1038/s41598-023-49219-6 PMC10724261

[8] Ko H Yan M Gupta R , et al. Predictors of survival in patients with advanced gastrointestinal malignancies admitted to the intensive care unit. Oncologist. 2019;24(4):483-490. 30518614 10.1634/theoncologist.2018-0328 PMC6459253

[9] Soares M Caruso P Silva E , et al. Characteristics and outcomes of patients with cancer requiring admission to intensive care units: A prospective multicenter study. Crit Care Med. 2010;38(1):9-15. 19829101 10.1097/CCM.0b013e3181c0349e

[10] Anisoglou S Asteriou C Barbetakis N , et al. Outcome of lung cancer patients admitted to the intensive care unit with acute respiratory failure. Hippokratia. 2013;17(1):60-63. 23935346 PMC3738280

[11] Wu M Gao H . A prediction model for in-hospital mortality in intensive care unit patients with metastatic cancer. Front Surg. 2023;10:992936. 36793319 10.3389/fsurg.2023.992936 PMC9922743

[12] Byrd TF 4th Southwell B , et al. Validation of a proprietary deterioration Index model and performance in hospitalized adults. JAMA Netw Open. 2023;6(7):e2324176. 10.1001/jamanetworkopen.2023.24176 PMC10366696 37486632

[13] Singh K Valley TS Tang S , et al. Evaluating a widely implemented proprietary deterioration Index model among hospitalized patients with COVID-19. Ann Am Thorac Soc. 2021;18(7):1129-1137. 33357088 10.1513/AnnalsATS.202006-698OC PMC8328366

[14] Gallo RJ Shieh L Smith M , et al. Effectiveness of an artificial intelligence-enabled intervention for detecting clinical deterioration. JAMA Intern Med. 2024;184(9):1137. 10.1001/jamainternmed.2024.0084 PMC10964159 38526472

[15] Mou Z Godat LN El-Kareh R Berndtson AE Doucet JJ Costantini TW . Electronic health record machine learning model predicts trauma inpatient mortality in real time: A validation study. J Trauma Acute Care Surg. 2022;92(1):74-80. 34932043 10.1097/TA.0000000000003431 PMC9032917

[16] Vincent JL Moreno R Takala J , et al. The SOFA (sepsis-related organ failure assessment) score to describe organ dysfunction/failure. On behalf of the working group on sepsis-related problems of the European society of intensive care medicine. Intensive Care Med. 1996;22(7):707-710. 8844239 10.1007/BF01709751

[17] Daviaud F Grimaldi D Dechartres A , et al. Timing and causes of death in septic shock. Ann Intensive Care. 2015;5(1):16. 26092499 10.1186/s13613-015-0058-8 PMC4474967

[18] Timsit JF Fosse JP Troché G , et al. Accuracy of a composite score using daily SAPS II and LOD scores for predicting hospital mortality in ICU patients hospitalized for more than 72h. Intensive Care Med. 2001;27(6):1012-1021. 11497133 10.1007/s001340100961

[19] Guiguet M Blot F Escudier B Antoun S Leclercq B Nitenberg G . Severity-of-illness scores for neutropenic cancer patients in an intensive care unit: Which is the best predictor? Do multiple assessment times improve the predictive value? Crit Care Med. 1998;26(3):488-493. 9504577 10.1097/00003246-199803000-00020

[20] Larché J Azoulay E Fieux F , et al. Improved survival of critically ill cancer patients with septic shock. Intensive Care Med. 2003;29(10):1688-1695. 13680115 10.1007/s00134-003-1957-y

[21] Driessen RGH Heijnen NFL Hulsewe RPMG , et al. Early ICU-mortality in sepsis - causes, influencing factors and variability in clinical judgement: A retrospective cohort study. Infect Dis (Lond). 2021;53(1):61-68. 32930619 10.1080/23744235.2020.1821912

[22] Steitz BD McCoy AB Reese TJ , et al. Development and validation of a machine learning algorithm using clinical pages to predict imminent clinical deterioration. J Gen Intern Med. 2023;39(1):27-35. 37528252 10.1007/s11606-023-08349-3 PMC10817885

[23] Edelson DP Churpek MM Carey KA , et al. Early warning scores with and without artificial intelligence. JAMA Netw Open. 2024;7(11):e2448969. 10.1001/jamanetworkopen.2024.38986 PMC11544488 39405061

[24] Cummings BC Blackmer JM Motyka JR , et al. External validation and comparison of a general ward deterioration Index between diversely different health systems. Crit Care Med. 2023;51(6):775-786. 36927631 10.1097/CCM.0000000000005837 PMC10187626

[25] Hui D dos Santos R Chisholm G , et al. Clinical signs of impending death in cancer patients. Oncologist. 2014;19(6):681-687. 24760709 10.1634/theoncologist.2013-0457 PMC4041673

[26] Jansen G Entz S Holland FO , et al. A comparison of simplified acute physiology score II and sepsis-related organ failure assessment score for prediction of mortality after intensive care unit cardiac arrest. Minerva Anestesiol. 2024;90(5):359-368. 38656085 10.23736/S0375-9393.24.17825-X

[27] Fayed M Patel N Angappan S , et al. Sequential organ failure assessment (SOFA) score and mortality prediction in patients with severe respiratory distress secondary to COVID-19. Cureus. 2022;14(7):e26911. 10.7759/cureus.26911 PMC9290429 35865183

[28] Antonelli M Moreno R Vincent JL , et al. Application of SOFA score to trauma patients. Sequential organ failure assessment. Intensive Care Med. 1999;25(4):389-394. 10342513 10.1007/s001340050863

[29] Kashyap R Sherani KM Dutt T , et al. Current utility of sequential organ failure assessment score: A literature review and future directions. Open Respir Med J. 2021;15(1):1-6. 34249175 10.2174/1874306402115010001 PMC8227444

[30] Nates JL Cárdenas-Turanzas M Wakefield C , et al. Automating and simplifying the SOFA score in critically ill patients with cancer. Health Informatics J. 2010;16(1):35-47. doi: 10.1177/1460458209353558 20413411

